# Developing a virtual reality (VR) application for practicing the ABCDE approach for systematic clinical observation

**DOI:** 10.1186/s12909-023-04625-2

**Published:** 2023-09-05

**Authors:** Helen Berg, Ekaterina Prasolova-Førland, Aslak Steinsbekk

**Affiliations:** 1 Department of Health Sciences, Norwegian University of Science and Technology, Ålesund, Norway; 2 Department of Education and Lifelong Learning, Norwegian University of Science and Technology, Trondheim, Norway; 3 Department of Public Health and Nursing, Norwegian University of Science and Technology, Trondheim, Norway

**Keywords:** Virtual reality, ABCDE approach, Self-practice, Simulation, Clinical skills

## Abstract

**Background:**

The Airways, Breathing, Circulation, Disability, Exposure (ABCDE) approach is an international approach for systematic clinical observation. It is an essential clinical skill for medical and healthcare professionals and should be practiced repeatedly. One way to do so is by using virtual reality (VR). The aim was therefore to develop a VR application to be used by inexperienced health students and professionals for self-instructed practice of systematic clinical observation using the ABCDE approach.

**Methods:**

An iterative human-centred approach done in three overlapping phases; deciding on the ABCDE approach, specifying the requirements, and developing the application.

**Results:**

A total of 138 persons were involved. Eight clinical observations were included in the ABCDE approach. The requirements included making it possible for inexperienced users to do self-instructed practice, a high level of immersion, and a sense of presence including mirroring the physical activities needed to do the ABCDE approach, allowing for both single and multiplayer, and automatic feedback with encouragement to repeat the training. In addition to many refinements, the testing led to the development of some new solutions. Prominent among them was to get players to understand how to use the VR hand controllers and start to interact with the VR environment and more instructions like showing videos on how to do observations. The solutions in the developed version were categorised into 15 core features like onboarding, instructions, quiz, and feedback.

**Conclusion:**

A virtual reality application for self-instructed practice of systematic clinical observation using the ABCDE approach can be developed with sufficient testing by inexperienced health students and professionals.

## Background

There is a need for medical and healthcare students to learn the Airways, Breathing, Circulation, Disability, Exposure (ABCDE) approach [1–4]. This is an international approach for systematic clinical observation that can be used in a range of situations, from acute cases to baseline registration in stable cases. The ABCDE approach and systematic clinical observation include specific clinical skills often learned using simulation methods.

Simulation is a pedagogical method based on active learning theory which provides the possibility to practice clinical skills in safe and controlled environments [5]. Simulation is defined as the imitation or representation of one act or system by another [6]. Studies show that simulation training has strong educational effects [7] and repetitions are regarded as highly important in simulation [8]. However, traditional physical simulation is a resource-demanding activity that can limit its use [9, 10].

Virtual Reality (VR) simulation offers an alternative to traditional simulation, and it has begun to be used in medical and healthcare education [11]. VR simulation can be performed in a three-dimensional (3D) virtual environment. In a 3D virtual environment, the player is represented by an avatar and can interact with the environment and other players in real-time from a desktop PC or using VR goggles (head-mounted display) [12]. Virtual Reality has been extensively used in professional training, including medical training [13], following the experiential [14] and constructivist learning approach [15].

Research shows both strengths and limitations with the use of VR technology [16–19]. An obvious advantage is the independence of geographical location, the opportunity to provide integrated feedback for self-practice, and that the students think it is fun and enthralling. Another advantage is that multiplayer versions of VR can potentially provide effective simulation-based group learning [20, 21]. Studies have shown that students practicing in a group learn from observing and helping each other when practicing clinical skills [22]. The arguments related to limitations are mainly technical challenges regarding the use of the software and some users becoming nauseous and dizzy (experiencing cybersickness) when using the VR goggles although considerably less so with improved equipment. But there are also challenges related to human perception and immersive adherence, indicating that the VR environments have to be recognizable and authentic [12].

We have not been able to identify any publication on the development of VR applications for practicing the ABCDE approach in a single or multiplayer fully immersive and interactive environment using head-mounted devices. Thus, the aim was to develop a virtual reality application to be used with commercially available VR goggles and hand controls by inexperienced health students and professionals for self-instructed practice of systematic clinical observation using the ABCDE approach.

## Methods

### Design

An interactive human-centred design method for interactive systems was used [23], as this approach focuses specifically on making systems usable and includes elements of agile software development methodology [24].

The overall approach included two main activities. Deciding on the clinical observations to be included in the application to ensure a focus on the order of observations of the ABCDE approach, and testing of the VR application at different stages of the development. This included specifying the requirements and testing specific functions of the VR application and finalising the application in an interplay between the researchers and programmer. The authors’ previous experiences from work with VR, simulation, and interprofessional education were used to make the initial version.

The game engine used for the final programming was Unity2018.3.0f2, which allowed for rendering to different commercially available VR equipment like Oculus Rift and Oculus Quest [25, 26], and HTC VIVE [27]. The work presented here was part of a research project investigating the effect of individual and group training in VR compared to practicing with physical equipment in two randomised controlled trials (RCTs) using the application presented here [19, 28]. A total of 578 first-year medical and nurse students participated, and it was found that the effect of the VR application was non-inferior to practicing with physical equipment .

### Background for the development processes

The intended overall learning outcome was that after using the ABCDE VR application, the learner had the knowledge of which important clinical observations are included in the ABCDE approach and further, had the skills to carry out ABCDE observations in the correct order and document the observations.

The primary target audience was medical- and healthcare students at the beginning of their first semester and health professionals inexperienced in the ABCDE approach and VR. To arrange for self-practice in VR it was deemed critical to make the application as intuitive as possible and suitable for players with no or little experience with the use of head-mounted VR equipment and hand controllers. The premise to achieve this was that the different elements in the VR application had to be simplified as much as possible to reduce distractions and cognitive load.

The reason for choosing VR to practice a procedural skill like the ABCDE approach is the advantage of practicing this more realistically than on a computer or tablet. This includes high-level immersion and sense of presence [29], and the possibility of doing the physical movement needed to perform the ABCDE approach in the real world. One example is that in VR the player has to move their physical hand to place their virtual hand on the arm of the virtual patient and they can feel the heartbeat through the hand controller.

To enhance the experience of reality in the VR application, both immersion (the technological quality) and sense of presence (the psychological experience) had to be considered [29–31]. Immersion is crucial for the player to start to feel in a real environment [32], while a sense of presence can be increased by integrating additional sensorial experiences in addition to vision, such as sound, the possibility to interact with the environment through touch, and haptic feedback from the hand controller [12]. Using a first-person perspective, where the environment is seen through the eyes of the player, helps to increase a sense of presence [30].

Skills like the ABCDE approach can be practiced both individually and in a group. Thus, the application was made to allow for both single and multiplayer. In multiplayer solution, it is recommended to take into account that the players are social actors where the possibility to interact with other people gives a sense of being a part of what’s going on [33]. Furthermore, every player must have something to do; if not, they are likely to lose focus [34].

Experiential learning is incorporated in the interactive VR nature, where the player can try, reflect and retry, in a continuing reconstruction of experience [35]. Furthermore, as studies show that a rapid loss of skills occurs after initial training [36, 37], providing repeated practice, which has been found to lead to the retention of skills among all types of healthcare professionals, is helpful for the learning outcome [38, 39]. As it was expected that practicing the same skill several times is needed to learn the ABCDE approach [40], motivation to repeat the practice was seen as important both within the application and in making the application easy and interesting to use again later on.

Feedback is frequently highlighted as being central to the learning process and motivates repetition [8, 41]. Feedback can be conceptualised as information provided by someone or something on aspects of one’s performance or understanding and is one of the most effective drivers of learning [42]. VR gives the possibility to provide an assessment with guidance and feedback [43] as it is possible to automatically register what the player does. This can be used to give an overall assessment in form of stars or grades and instant feedback on the actions done.

### Participants

For deciding on the ABCDE approach, the aim was to include professionals from the healthcare practice field and academia with experience with the ABCDE approach either clinically or as teachers. They were identified by searching courses for ABCDE or systematic clinical observation skills, and through the author’s professional network.

For testing the application during development, the aim was to include persons with a range of different backgrounds including students and professionals from different healthcare disciplines as well as persons without such knowledge. This was done to ensure that the VR application could be used without any previous interest or knowledge in health sciences or VR. Participants were recruited opportunistically by the first and last author who asked persons connected to healthcare education, attending meetings and seminars, and whom they had chance meetings with.

### Data collection

The data was collected from February 2018 to May 2019.

To collect the data needed to decide on the observations to be included in the ABCDE approach, literature and web pages describing the ABCDE approach were identified. The findings were then presented and discussed with participants holding clinical and pedagogical competence. They were asked what they saw as the most important observations to include, given that the learning objective was that inexperienced learners should learn the order of the ABCDE approach and not the details of each observation.

To collect data for the development of the application, data from the participants’ experiences from trying the different versions of the application were collected through notes taken during observation and answers to questions given orally. In addition, the communication between the domain experts (the authors) and the hired programmer, was collected.

### Analysis

The analysis of the data was supported using software (NVIVO 12 and Mindjet MindManager 2019).

For deciding on the ABCDE approach, findings from the literature and comments from the participants were assessed by the authors and informed the decision on which features to be included in the VR application. After several iterations, a final suggestion was presented to a group of professionals from the healthcare practice field and academia and accepted.

For developing the initial version of the application, the comments of the participants from the iterative testing were discussed in weekly meetings between the authors and the hired programmer. Based on these comments, modifications were decided on and revised versions were tested subsequently.

## Results

A total of 18 persons from the healthcare practice field (n = 9) and academia/teaching institutions (n = 9) participated in providing input on the observations to be included in the ABCDE approach.

The UK resuscitation guidelines [4] were chosen as the starting point for selecting the clinical observations to be included in the application. The result was eight basic ABCDE observations (Table 1). The argument for the selection was to include the observations used for early detection of worsening somatic conditions in healthcare wards [44] and in conducting a primary ABCDE observation (i.e., omitting secondary observations) [4].

**Table 1:** The eight observations selected to practice the ABCDE approach in the order they were practiced and with description of the examination method used for each observation

Area	Observation
Airways	A: Free or obstructed airways by inspecting the mouth cavity
Breathing	B1: Respiratory frequency by counting chest movement, listening to respiration sounds or feeling vibration in the hand controller placed on the chest.
	B2: O2 Saturation measured using a digital pulse oximeter
Circulation	C1: Blood pressure measured using a digital blood pressure gauge
	C2: Heart rate by counting the radial pulse presented as vibration in the hand controller placed on the lower arm
Disability	D: Awake or unconscious by inspecting the face mimic of the patient avatar
Exposure	E1: Body temperature measured using a digital ear thermometer
	E2: Normal or not normal skin by inspecting the colour of skin of the patient avatar

For the iterative testing and development of the application, a total of 120 persons participated in addition to the authors. Approximately 10 versions of the application were tested. Changes between the versions ranged from correcting technical errors, via adjustments like which angle to show the virtual hand in when different procedures were carried out, to a total remake of the onboarding process. The main solutions and features in the final application are presented in Table 2.

**Table 2:** Description of the features of the ABCDE VR application

Application features	Explanation
Setting	Be present in first-person in a 3-dimensional virtual wardroom, equipped with devices for clinical measurements, having a 360-degree vision.
Interaction	Virtual hands to pick up and move things, with haptic response in the controllers.
Onboarding	Making the players start interacting with the environment immediately. First by having to lift their hand to point with a laser. Then a tutorial to move objects and look around, starting from touching a ball and moving it, to performing one of the observations (measuring blood pressure) and documenting the results. Detailed oral and written instruction was given for all the tasks.
Multi-player cooperation	Online synchronisation for full interaction and audio and visual contact between the avatars of the group members. Avatar and player lip-synchronization and spatial sound coming from the direction of the speaker.
Activity for all the participating players	Question boards pop-ups related to the active observation going on. The questions concerned the meaning of the observation letter (e.g., ‘A’ for ‘Airways’) and the actual observation that should be done.
Rotation of positions in multiplayer	Players automatically rotated after one player had completed the ABCDE observation to give all players the possibility to do clinical observations.
Simplifying use of hand controllers and equipment for observations	Only one button on the hand controller was activated (grip button on the hand controller to point, pick up, hold, and release things). Teleportation was not possible. Before measurements of clinical values, it was sufficient to place the virtual equipment used to measure saturation, blood pressure and body temperature, in the (approximately) correct position (e.g., in the patient’s ear)
Virtual patient (VP)	A healthy older male person lying on the bed half-dressed, having a visual response (eye blinking, head movement, open and close mouth, chest movement), haptic response (breath, pulse on the wrist), and randomly changing clinical value responses to be measured by using virtual equipment (blood pressure, temperature, oxygen saturation).
Haptic feedback	Pulse in the hand controllers when feeling the pulse (each heartbeat) on the wrist, and when placing the hand on the chest (each respiratory intake) and feedback on the touch on the documentation tablet and the patient monitor.
Audio feedback	Inflation sounds from the blood pressure gauge and “bip” from the ear thermometer when the measure is ready (5 s).
Wristwatch	On left hand. Classic design showing real-time including seconds.
Patient monitor	Monitor with touch screen buttons to get clinical values (blood pressure, temperature, oxygen saturation).
Documentation tablet	Tablet with touch screen buttons for documentation of the observations, including numeric pad for entering clinical values and choice between predefined options.
Instructions for self-practice	A silent subtitle video running on a wall-mounted screen showing how to do the observations when they were selected by the player, and a poster on the wall with the ABCDE observations. Everything that the player could interact with, was marked with a yellow circle.
Feedback	When the player selects that all documentations are done, a scoreboard appears with detailed feedback and a summary maximum of three stars, covering the order of observations, whether all observations were done and if the values from the observations were correct.

The most prominent change to the initial solution, which was made before the testing began in earnest, concerned onboarding into the application, the instruction and guidance given during the play, and the type of equipment available. These solutions were added as some players either could not start or proceed or used a long time to figure out what to do. The situations that encountered the most problems and the solutions for these are described in some detail below.

It was frequently observed that players did not use the controllers as intended, especially when the use of more than one button was an option. This was simplified so that only one button (grip button in front of the controller) could be used. This meant the solutions which required the use of more buttons on the controller, e.g., to move in the room through teleportation, was removed. This meant that the player’s movement in the virtual room was restricted by the physical area the player could move within, and in practice, they moved along the bed with the patient and the table/wall with the equipment to do the observations.

A major challenge was the onboarding process where some players did not use their hands once they had entered the application. They seem not to be aware of their hands as they could only see them if they lifted their hand. It took much trial and error to make all subsequent players aware that they had hands and that they could use them to interact with the environments. The final solution included two features for onboarding (Table 2). First, when the players put on the gear, they saw an instruction asking them to select single or multiplayer with a video showing hands being lifted with a laser beam pointing towards the two choices. There was also a rotating hand with the laser beam in front of the players that highlighted the front button on the hand controller they had to press.

However, this was not enough onboarding practice for some players as they did not go on to observe or interact more actively with the environment. This was a problem, as to complete all observations, they had to use both hands, gripping, moving, and releasing objects. The solution became adding a tutorial starting immediately after choosing single or multiplayer. This part started at a very basic level and moved to more complex tasks. First, they were asked to touch a red ball that hovered in front of them without any other instruction (the player’s view based on their height was also adjusted automatically by this). Then the tutorial progressed to ask the players to grip the red ball and hold it using the grip button and move it to a circle that was outside their field of vision to get them to move their heads. This was achieved with an arrow pointing in the right direction. All players followed these instructions and thus become aware of their hands and the need for turning their heads to look around. After this, the initially planned instruction on how to use equipment and document clinical values commenced. This included using the blood pressure gauge that players had to place on the patient’s arm, pressing a button on a monitor to get the clinical value, and documenting the result on a tablet by using their hand that transformed into a hand with a pointing finger to enter numbers using a numeric keypad. It was found that it was also necessary to guide this part with voice and film instructing the players on what to do.

**Fig. 1:**
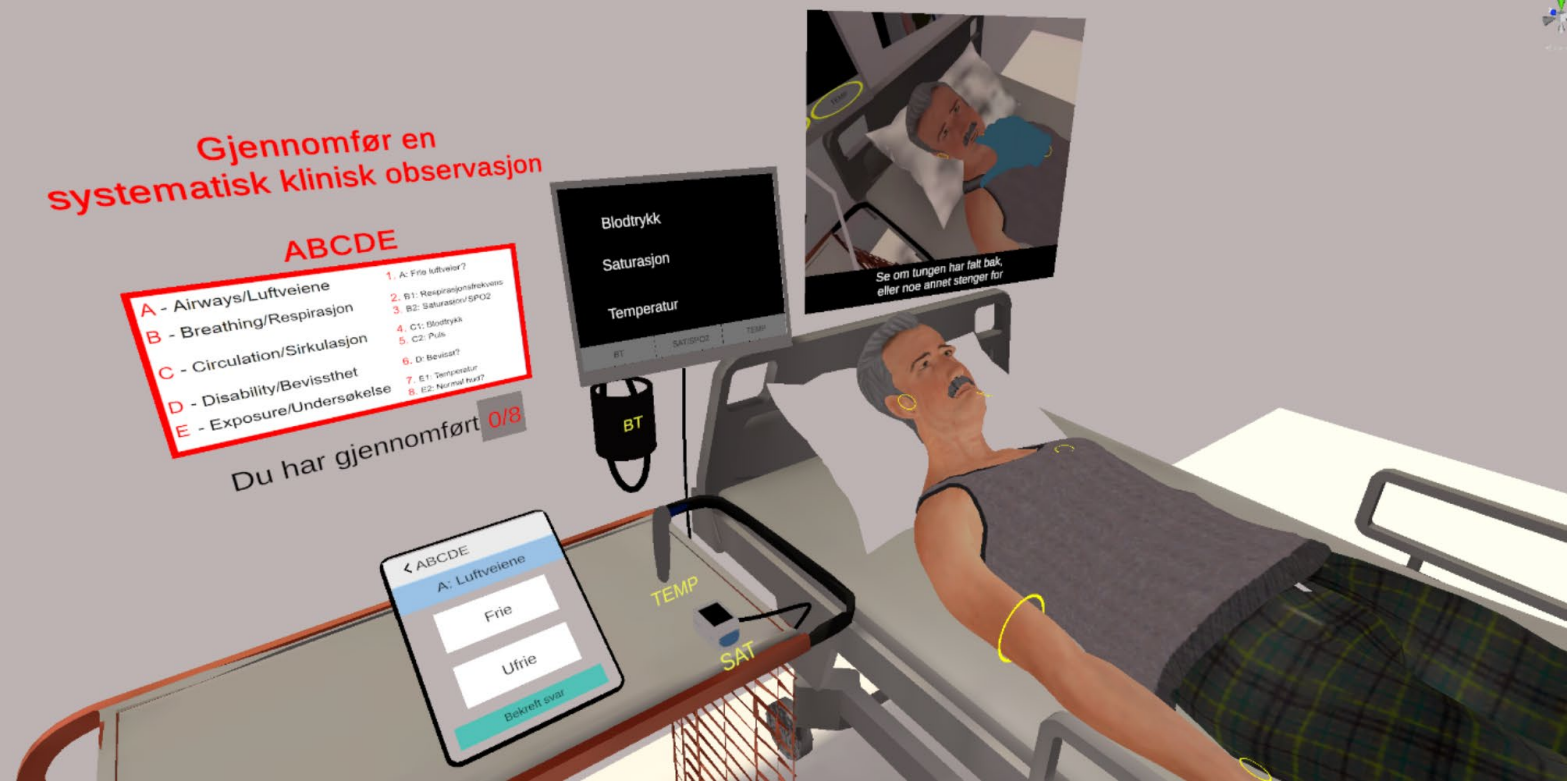
The ABCDE applications virtual wardroom shown from the view of the player doing observations. The equipment that could be used are marked with yellow. The tablet used to document the clinical values is to the left on the table. The monitor displaying values from the digital equipment is in the middle of the picture, with hoses connected the gauges. The guidance film is in the upper right corner. On the wall above the tablet was the instructions on what to do and the order of the observations in red

Several adjustments had to be done to ease the use of the equipment needed for observation to avoid cognitive load from too much focus on the technical aspect of doing the observations. One example was measuring blood pressure. Testing revealed that measuring manually was too complex as it involved inflating the cuff by pressing one button to increase pressure and another to decrease pressure. It was therefore chosen to use a digital blood pressure gauge with functions as described in Table 2. To make it intuitive to touch the monitor, a hose was animated from the blood pressure gauge to the monitor (Fig. 1).

To help the player to understand the possibilities of interactions and where to do the clinical observation on the avatar patient, yellow circles were placed on everything the player could interact with. To further guide the player, a film on a wall screen showed how to do the observation after they had chosen which observation to do. The film ran in a loop until a new observation was chosen (Fig. 1).

To avoid distractions and reduce cognitive load, the observation was done on a patient in a stable condition with random variation in the clinical values within the normal range of a healthy elderly person. Also, a simplified wardroom was used, without other equipment than the ones used for the observations. This was done as testing of early versions of the application showed that players were, for example, distracted by a mirror where they could see their avatar, the possibility to look out of a window, and the possibility to teleport.

**Fig. 2:**
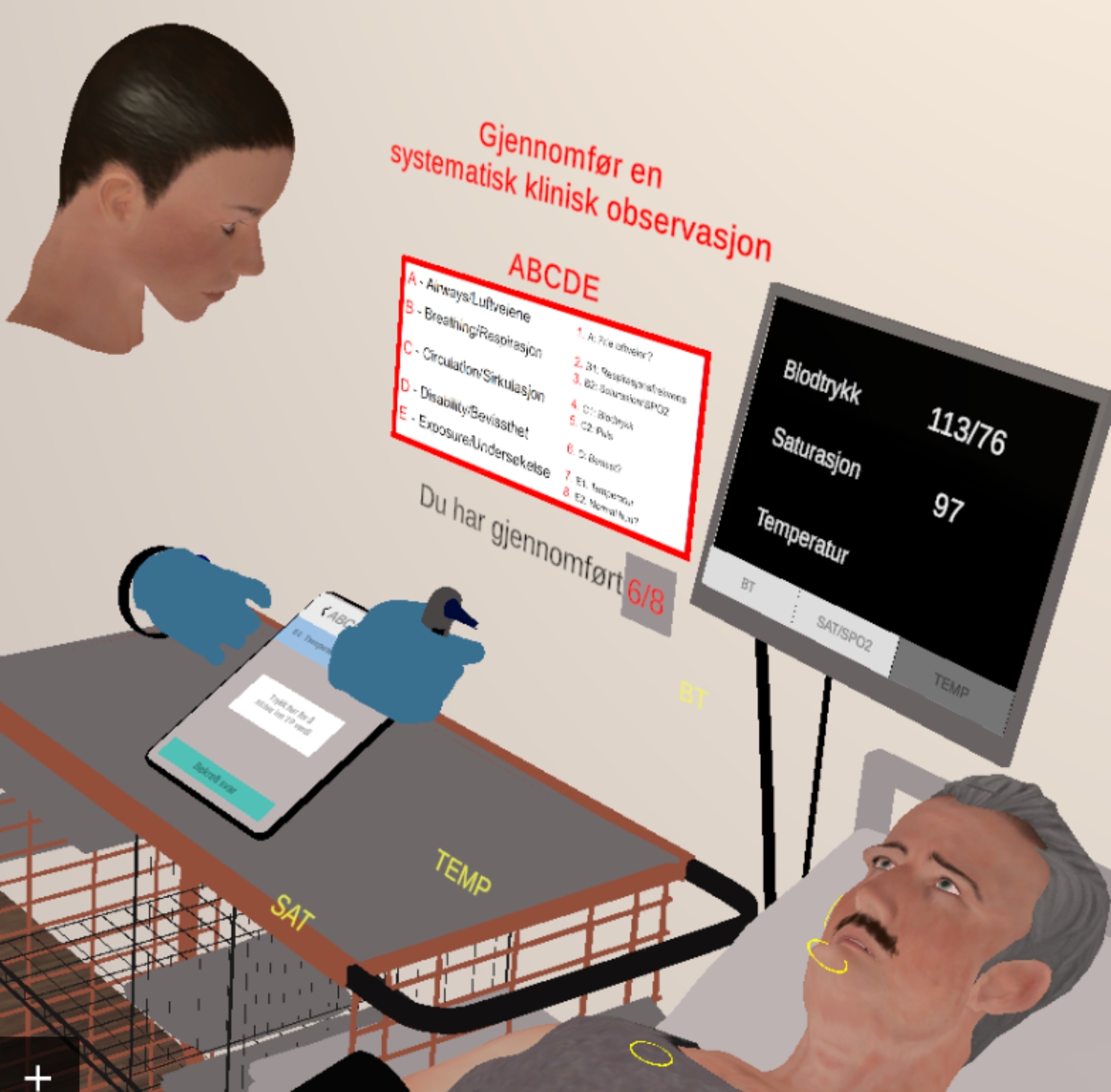
A player doing an observation seen from the viewpoint of another player in multiplayer mode. The avatar measures the patient’s temperature, holding the digital thermometer in the right hand. When the thermometer is placed in the ear, a bip sounds after a few seconds indicates that the measurement is ready and that the button on the monitor (currently grey) can be pressed to get the clinical value

When testing the first versions of the multiplayer version, it was obvious that the two players observing and helping the player who carried out the ABCDE examination lost focus when e.g., pulse was taken as this involved a long stretch of inactivity and silence. A pop-up quiz tablet was introduced to help all players refocus on what was going on. The quiz tablet appeared every time the player in the active position started on a new observation. It was observed that this helped to catch the attention of the others and engage them more in the ongoing observations. The quiz tablet had questions about which observation to do and gave instant feedback on whether the answer was correct or not. The testing showed that having full-bodied avatars representing the players without using body sensors on the player meant that the avatar had some strange movements. It was tested to use player avatars with only heads and hands, and the tests showed that this was acceptable. When asked, players commented that they did not give it a second thought that the avatar did not have a body. The automatic patient avatar was full-bodied (Fig. 2).

**Fig. 3:**
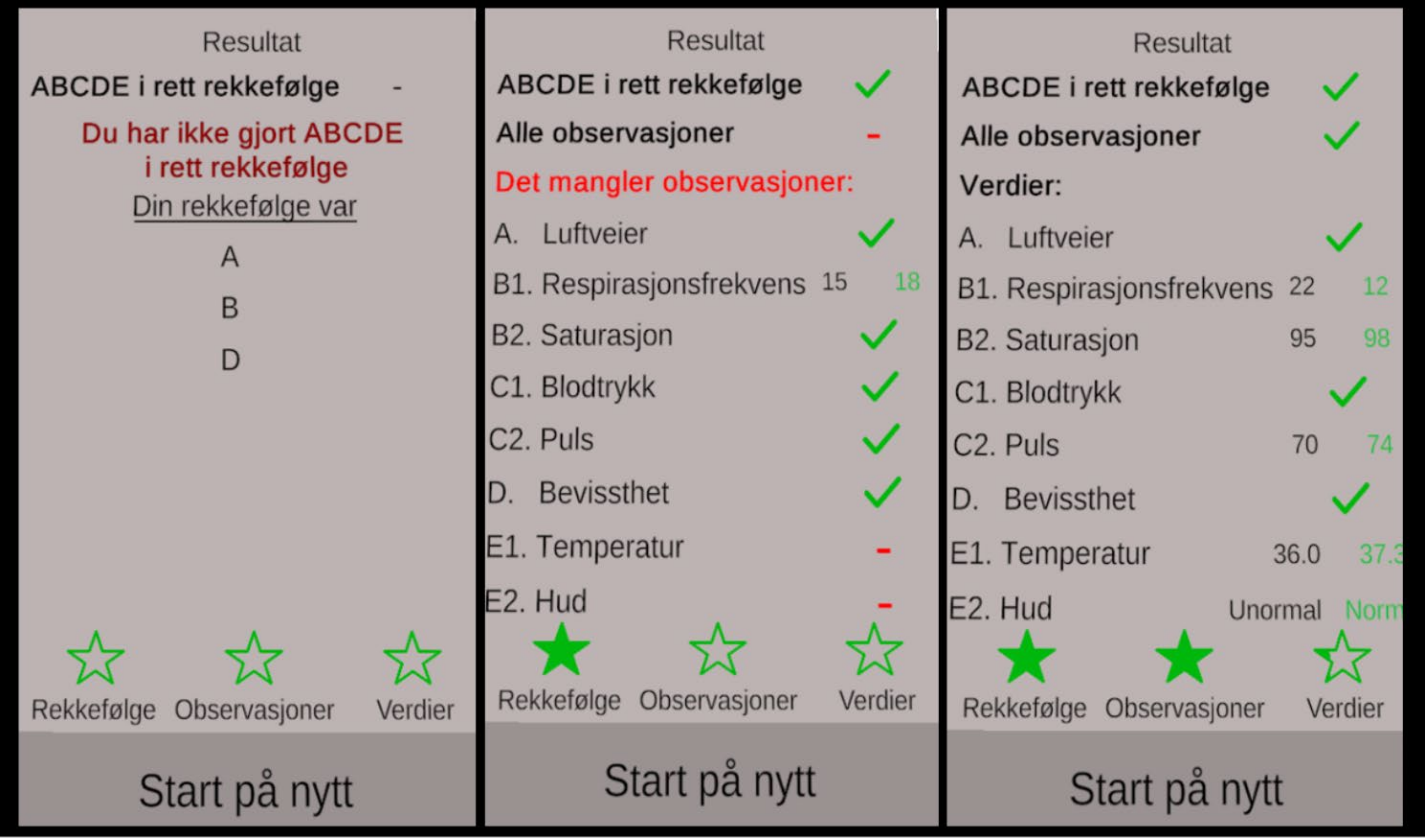
The feedback tablet after completion. The left column shows an example with all missing observations, and wrong order of the ABCDE. The middle shows some missing observations, but right ABCDE order. The right column shows all the learning goals have been achieved but some values have been measured incorrectly

In the first versions tested, the planned feedback was not implemented, and several players requested this spontaneously. They wanted to know if they had got the observations right. Different types of feedback were tested, aiming to balance the focus on (a) doing the observations in the right order, (b) the time needed to understand the feedback and the trustworthiness of the feedback, and (c) pointing to what needed to be focused on when repeating the exercise. The solution became a feedback pop-up board which was displayed after the player had decided that he/she was finished with the observations. The pop-up board gave feedback on whether the order of the ABCDE observations was correct, whether all observations were done, and if the clinical values documented by the player were correct (Table 3; Fig. 3). For each of these, a star at the bottom of the board was filled with a green colour if everything was correct, and partly filled if something was missing. After the feedback, the players got the choice to play again or quit, with the encouragement “practice makes perfect”.

**Table 3:** The features of the feedback on the pop-up board in the ABCDE VR application

Area	What was done
Feedback on achieved learning outcome	Presenting the results of the practice in the prioritized order of the learning outcomes, showing (1) if the order of the observations was correct, (2) which observations was done and (3) if the clinical values observed were correct.
Allowing enough time to understand feedback	Presenting one item at a time. Marking as correct or incorrect. Summarise with three stars the three areas on which feedback was given (see above).
Pointing out areas for improvement	Marking with colours (green and red) whether the results of the practice were completed successfully or not.

The usability of this application was tested in two randomized controlled trials (RCT), one single- and one multiplayer, both showing similar usability as practicing the ABCDE approach with physical equipment [19, 28].

## Discussion

To ensure focus on the ABCDE order, eight observations were agreed upon. Most of the requirements to ensure immersion, presence, interaction, and self-practice were prespecified, but several solutions had to be added during the development process based on user feedback. The most important were the ones related to the onboarding process to get players to understand how to use the VR hand controllers and start to interact within the VR environment by adding more introductions.

A strength in this study was the involvement of students from various educations and professionals both from healthcare and outside to assure both the clinical relevance and the software usability, and the extensive testing including the investigation of the effect in two randomised controlled trials [19, 28]. It is a limitation that personal information about the participants was not collected. Although intended, the strong focus on making the application usable for persons inexperienced with VR and/or the ABCDE approach means that its use is limited to a basic introduction. Other solutions are needed for practicing advanced ABCDE.

The testing done in this study and the results from the two RCTs conducted to test the effect of the application [19, 28], confirmed that players inexperienced in the ABCDE approach and in using VR were able to do the observations and document these only by using the application. This makes it possible to use the application for self-practice without additional teaching resources involved. It also means that the application can be suited to refresh ABCDE skills repeatedly over time as players can use it even if they have not used VR regularly.

Nevertheless, this simplified ABCDE application is adapted for beginners, due to the restricted number of observations and simplified way of doing the observations. As soon as the players get familiar with both the VR technology and the ABCDE order, the need to advance the learning requires an advanced ABCDE application. One way to develop a more advanced version is by adjusting the tutorial/guidance to the level of the player and adding more options, scenarios, and ‘game levels’ for the player to be able to handle more varied and severe clinical cases.

The findings support the existing research on user interaction and what tasks are suitable for training in VR and what is not. Bassano et al. (2018) argue that VR is suitable for familiarizing with the environment and procedures, but not for how to use a certain tool [45]. In the case described in this paper, this translates to focusing on the order of the observations and not the details of how to do the observations themselves in real life. To be able to do more advanced tasks and observations, support for fine motoric operations, which is not possible with today’s hand controllers, would be needed. With the development of tracking finger movement, this becomes possible, ideally with the possibility of haptic feedback.

In a study on serious games, the researchers concluded that having high fidelity (many features) in the game produced too high a cognitive load [46]. Cognitive overload is a situation where learning is not achieved due to the amount of things happening, which leads to an overburden of the working memory [47]. A lesson learned during this current study was to reduce cognitive load by simplifying technical procedures and reducing elements that could take the player’s focus away from the learning goal (to learn the ABCDE order). In a study on repeated in-situ simulation to practice the ABCDE approach, it was found that the teams did not follow through on the systematic ABCDE approach throughout the whole simulation [48]. The suggested reason was that the clinical cases shifted between each simulation, and many of the professionals were not familiar with the ABCDE approach, or with simulation as a training method. It has been suggested to move from simple to more complex situations by moving from low to high-fidelity environments [49]. The argument would be that the competency is built up gradually by reducing the initial load [50, 51].

It was decided during the development process to do as much simplification as possible, e.g., by only including the features the players needed to do the clinical observations, and not use full body avatars. Based on the feedback from the participants in the two RCTs [19, 28] and testers in this study, the reality of the patient ward was recreated in the virtual reality environment even if it was reduced. A likely reason is that the application turned out to be both immersive and interactive which is not seen in other solutions [11, 20, 52].

The ABCDE approach is a skill that must be automated to be used in a critical situation. Thus, repeated practice is needed [11, 53, 54]. This was the main motivation for the development of the VR application presented here. A review showed that self-regulated learning in simulation could lead to improved and good learning outcomes [55]. Although creating an application for self-practice was intended from the start, the testing showed that more feedback was needed. Feedback is, pursuant to Hattie and Timperley (2007) one of the most effective drivers of learning [42]. Other studies have found feedback to be the single most important feature in simulation-based learning with regard to the goal of effective learning [8], and positive and targeted feedback gave better learning and performance outcomes [22].

A take-home message from this study was that doing the testing on persons not familiar with VR was important. This is the reason for the high number of testers (N = 120), which is far beyond what is usually the case [56, 57]. It was found helpful to use testers inexperienced with VR.

## Conclusion

It was feasible to develop a virtual reality application to be used by inexperienced persons for self-instructed introduction to practice basic systematic clinical observation using the ABCDE approach. The involvement of many end-users at different stages of the development was essential to ensure that the time is spent on the main task and not on learning to use VR. To develop an application for self-practice among inexperienced users, testing on a high number of novices to VR was found to be fundamental.

## Data Availability

The datasets analysed during the current study are available from the corresponding author upon reasonable request.

## List of abbreviations

ABCDE: Airways, Breathing, Circulation, Disability, Exposure
VR: Virtual Reality
3D: Three-dimensional
RCT: Randomised controlled trial
